# 20:4-NAPE induced changes of mechanical sensitivity and DRG neurons excitability are concentration dependent and mediated via NAPE-PLD

**DOI:** 10.1038/s41598-025-98567-y

**Published:** 2025-04-23

**Authors:** Anirban Bhattacharyya, Daniel Vasconcelos, Diana Spicarova, Jiri Palecek

**Affiliations:** Laboratory of Pain Research, Institute of Physiology of the Czech Academy of Sciences, Prague, Czech Republic

**Keywords:** 20:4-NAPE, NAPE-PLD, Anandamide, CB_1_, TRPV1, DRG neurons, Sensory processing, Neuroscience, Pain

## Abstract

Alterations in the excitability of dorsal root ganglion (DRG) neurons are critical in the pathogenesis of acute and chronic pain. Neurotransmitter release from the terminals of DRG neurons is regulated by cannabinoid receptor 1 (CB_1_) and transient receptor potential vanilloid 1 (TRPV1), both activated by anandamide (AEA). In our experiments, the AEA precursor N-arachidonoylphosphatidylethanolamine (20:4-NAPE) was used to study the modulation of nociceptive DRG neurons excitability using K^+^-evoked Ca^2+^ transients. Intrathecal administration was used to evaluate in vivo effects. Application of 20:4-NAPE at lower concentrations (10 nM − 1 µM) decreased the excitability of DRG neurons, whereas the higher (10 µM) increased it. Both effects of 20:4-NAPE were blocked by the N-acylphosphatidylethanolamine phospholipase D (NAPE-PLD) inhibitor LEI-401. Similarly, lower concentrations of externally applied AEA (1 nM − 10 nM) inhibited DRG neurons, whereas higher concentration (100 nM) did not change it. High AEA concentration (10 µM) evoked Ca^2+^ transients dependent on TRPV1 activation in separate experiments. Inhibition of the CB_1_ receptor by PF514273 (400 nM) prevented the 20:4-NAPE- and AEA-induced inhibition, whereas TRPV1 inhibition by SB366791 (1 µM) prevented the increased DRG neuron excitability. In behavioral tests, lower 20:4-NAPE concentration caused hyposensitivity, while higher evoked mechanical allodynia. Intrathecal LEI-401 prevented both in vivo effects of 20:4-NAPE. These results highlight anti- and pro-nociceptive effects of 20:4-NAPE mediated by CB_1_ and TRPV1 in concentration-dependent manner. Our study underscores the complexity of endocannabinoid signaling in pain transmission modulation and highlights 20:4-NAPE as a potential therapeutic target, offering new insights for developing analgesic strategies.

## Introduction

The discovery of anandamide in 1992 (N-arachidonoylethanolamine, AEA), the first isolated endocannabinoid, a derivative of arachidonic acid synthesized in many peripheral tissues and organs as well as in central and peripheral nervous system led to early recognition of AEA as a critical mediator in many pathophysiological states including pain^1–3^. Even more than 30 years after the AEA identification, the mechanisms of AEA-mediated analgesia are still not fully understood. Extensive investigation, predominantly in rodent models, has brought exciting preclinical insights into AEA’s role in pathological pain conditions^4,5^. However, clinical trial failures of AEA-related drugs^6,7^ underscore the need for a deeper comprehension of AEA-induced effects, underlying mechanisms, and disparities between rodents and human physiology before advancing in clinical development. These disparities pose a major challenge for the translation of preclinical findings into effective clinical therapies, highlighting the need for more nuanced investigations into AEA’s mechanisms of action. Modulation of AEA metabolism is one of the challenges, and focusing on the AEA precursor 20:4-NAPE may be a novel approach to locally target the endocannabinoid system to elicit analgesia^8,9^.

Nociceptive signaling from the periphery is transmitted from primary sensory neurons (DRG neurons) to dorsal horn neurons. In the superficial spinal cord dorsal horn, there is abundant co-expression of two essential AEA targets, CB_1_ and TRPV1 receptors^10,11^. The vast majority of both these receptors are presynaptically localized on the central endings of DRG neurons, and their activation modulates excitatory neurotransmitter release from these sensory fibers. Meanwhile, the opposite regulation of glutamate release from DRG neurons by CB_1_ and TRPV1 receptors activation may occur at these first nociceptive synapses. Activation of G-protein coupled receptor CB_1_ decreases glutamate release via inhibition of high-voltage activated N- and P/Q-type Ca^2+^ channels and activation of inwardly rectifying potassium channels^12^. In comparison, the accumulation of Ca^2+^ ions in the central endings of DRG neurons, following activation of the Ca^2+^-permeable TRPV1 channels, represents the essential stimulus to trigger neurotransmitter release^13–15^. In particular, the application of exogenous AEA to spinal cord slices elicits a dual excitatory and inhibitory effect at these first nociceptive synapses^16,17^. However, peripheral inflammation altered this balance and shifted the AEA-induced responses predominantly toward neurotransmitter release inhibition^17^. These effects of externally applied AEA contrast with the apparent inhibitory effects of 20:4-NAPE application^18,19^. Together, these results suggest that the impact of AEA on nociceptive synaptic transmission may vary significantly depending on whether it is applied externally or synthesized locally from precursors by available enzymes and is further modulated under pathological conditions.

AEA is synthesized from membrane phospholipids primarily by the enzyme N-acylphosphatidylethanolamine phospholipase D (NAPE-PLD) when intracellular [Ca^2+^] increases during stimulation^19–21^. NAPE-PLD ensures the production of *N*-acylethanolamines (NAEs) from NAPEs or pNAPEs^22^. Moreover, AEA synthesis may occur through alternative anabolic Ca^2+^-insensitive pathways. Protein kinases (PK) A and C are implicated in this alternative AEA production in DRG neurons^23,24^. Subsequently, degradation of AEA into arachidonic acid and ethanolamine is rapidly ensured by fatty acid amino hydrolase (FAAH).

The objective of this work was to investigate the analgesic potential of various concentrations of 20:4-NAPE in vivo, using intrathecal administration and tactile sensitivity alteration assessment. The second objective was to study how 20:4-NAPE modulates the excitability of DRG neurons in vitro, compared to externally applied AEA in dissociated DRG cultures, with a particular focus on NAPE-PLD activity and CB_1_- and TRPV1-receptor-mediated mechanisms.

## **Materials and methods**

### Animals

Adult male Wistar rats (Institute of Physiology CAS, Czech Republic) were maintained at 22 ± 2 °C in light-controlled 12 h light/dark cycle conditions with free access to food and water. All experiments were approved by the local Institutional Animal Care and Use Committee and were carried out in accordance with the EU Directive 2010/63/EU for animal experiments, ARRIVE guidelines (https://arriveguidelines.org), the U.S. National Institutes of Health Guide for the Care and Use of Laboratory Animals, and guidelines of the International Association for the Study of Pain.

### DRG cell culture Preparation

DRG cells were isolated from adult male Wistar rats (approximately 200 g body weight) based on the previous protocol^25^ with modifications described in our previous work^26^. Briefly, rats were anesthetized deeply with Isofuran^®^, and intact L3-L5 DRGs (total 6 per animal) were dissected out bilateral to the spinal cord and collected in ice-cold DMEM (Invitrogen, #10566-016). The DRGs were then incubated in a pre-warmed enzyme cocktail containing 4 mg/ml Collagenase Type II (Invitrogen, #17101015) and 3 mg/ml (∼2 U/ml) Dispase II (Sigma-Aldrich, #D4693) for 45 min in a 37 °C incubator (oxygenated with 95% O_2_ and 5% CO_2_). The enzyme solution was washed out gently with fresh DMEM, and tissues were transferred to a 15 ml Falcon tube containing DMEM with 1% BSA and 1 mg/ml DNAase. DRG tissues were triturated with a glass Pasteur pipette in this solution using up and down strokes and mechanically dispersed. Digested and dispersed cells were filtered using a 40 μm Corning^®^ cell strainer (Sigma-Aldrich, #CLS431750) to obtain cell suspension devoid of undigested tissue debris and larger diameter (non-nociceptive) cells. Suspended DRG cells were then centrifuged at 200 × g for 5 min, washed by repeating the centrifugation step, and the obtained pellet was re-suspended in approximately 500 µl growth media containing 1:1 DMEM/F-12 (Invitrogen, #31331-028), 10% of Fetal Bovine Serum (FBS, Sigma-Aldrich), and 100 U/ml penicillin-streptomycin (Gibco, Thermo Scientific). 30–40 µl of DRG cell suspension was spotted onto 10 mm glass coverslips coated with poly-D-lysine (1 mg/ml) and laminin (0.02 mg/ml) and incubated for 2 h at 37 °C. The unattached and dead cells were washed out from the coverslips by adding fresh growth media after 2 h of plating, and the attached cells were kept in the incubator overnight at 37 °C. The cells were stained and imaged the next day, 18–24 h after). All procedures except animal dissection were performed in sterile conditions in a culture hood.

### Calcium imaging

Calcium imaging experiments were performed according to the established protocol^27^ with appropriate adjustments. Briefly, glass coverslips with attached DRG cells were incubated in artificial cerebrospinal fluid (ACSF) containing membrane permeable Ca^2+^ indicator dye Fura-2-acetoxymethyl ester (Fura-2 AM; Invitrogen, # F-1221) at a final concentration of 1 µM and Pluronic F-127 (Sigma-Aldrich) dissolved in DMSO at a final concentration of 0.01% for either 45 min in a 37 °C incubator or 1 h at room temperature in darkness. The ACSF solution contained 142 mM NaCl, 3 mM KCl, 2 mM CaCl_2_, 1 mM MgCl_2_, 10 mM HEPES, and 10 mM glucose adjusted to pH 7.4 with NaOH. Fura-2 incubated cells were washed 3 × in ACSF, and fluorescence images were captured in real-time under a 20 × water immersion objective using a Leica DM LB2 microscope (Leica Microsystems, Germany). Cells were exposed to alternating 340 and 380 nm wavelengths of excitation and emissions. The emitted light images were collected at 510 nm (TILL Photonics) during image recording at a frame rate of 1 Hz. Post-hoc image analysis was performed using MetaFluor^®^ software (Molecular Devices, USA) as a time-series experiment. The ratio of light intensity (340/380 nm) reflected changes in intracellular free Ca^2+^ concentration ([Ca^2+^]i) and was followed in ≥ 30 DRG cells simultaneously. During the experiment, cells were continually perfused with ACSF, in which all tested drugs where dissolved and applied focally to the cells under the field-of-vision using a custom gravity-driven fast perfusion system WAS-02^28^ attached with a fiber-glass application outlet of 0.1 mm diameter.

#### Experimental protocols

A high concentration of KCl (30 mM) was focally applied for 2 s repeatedly at 2 min intervals. The average amplitude of two K^+^-induced Ca^2+^ responses was calculated and set as a control value before drug treatment. Subsequently, different concentrations of 20:4-NAPE (10 nM, 100 nM, 1 µM, and 10 µM) and AEA (1 nM, 10 nM, and 100 nM) were administered for 6 min to evaluate the impact on K^+^-induced responses. Inhibition of K^+^ responses was calculated in % of control value before treatment. At the end of the experimental protocol, the TRPV1 agonist capsaicin (0.5 µM, 3 s) application was used to identify TRPV1-positive cells.

Inhibition of CB_1_ and TRPV1 receptors was tested using antagonists PF514273 (400 nM) and SB366791 (1 µM) pretreatment (4 min) followed by 6 min co-application with 20:4-NAPE (100 nM, 10 µM) and AEA (10 nM), respectively. The effect of the NAPE-PLD inhibitor LEI-401 (1 µM) was studied using DRG culture incubation (2 h) followed by acute application during the experimental protocol involving repetitive KCl (30 mM, 2s, 2-min interval) applications.

The effect of the high concentration of AEA (10 µM) was studied using a protocol consisting of 1st KCl (30 mM, 2 s) application followed by five consecutive applications of AEA (10 µM, 2 s, 2-min interval). When antagonists PF514273 (400 nM) and SB366791 (1 µM) were tested, the 6 min application of antagonists was used after the two short AEA applications.

### Behavioral testing

#### Electronic von Frey test

Adult male Wistar rats (250–300 g) used in experiments were housed with a 12 h light/12 h dark cycle and in standard conditions with food and drinking water available ad libitum. Rats were placed in clear plastic cages on a grid or glass plate and left to adapt to the testing environment for at least 20 min before any stimulation. Paw withdrawal threshold (PWT) to tactile stimulation was tested manually using the electronic von Frey apparatus (IITC Life Sciences, Model 2390 Series). The PWT was tested 4 times for each hind paw with at least 5 min intervals between the trials. The average value from each hind paw was calculated and then averaged in the experimental group. All procedures were based on our established behavioral tests^29–31^. The baseline withdrawal threshold was acquired at the beginning of each experimental protocol before drug administration.

#### Design of experiments

In the first series of experiments, a single i.t. application of four different concentrations of 20:4-NAPE (0.2 µM, 2 µM, 20 µM, and 200 µM) corresponding to the dose of 2 ng, 20 ng, 0.2 µg, and 2 µg per animal in a volume of 10 µl followed by 35 µl of saline was made in combination with in vivo measurement of PWT and PWL before and 1 h, 2 h, and 4 h after i.t. treatment. Rats received i.t. 20:4-NAPE from the lowest to highest concentration, and a two-day interval was maintained between the individual experiments.

In the second series of in vivo experiments, individual animals were tested for the baseline setting. Rats underwent behavioral testing 1 h after i.t. pretreatment with LEI-401 (10 µM, equal to the dose of 42 ng per animal) in the volume of 10 µl followed by 35 µl of saline. Subsequently, i.t. co-administration of LEI-401 (10 µM, 10 µl) and 20:4-NAPE (20 µM or 200 µM, 10 µl) followed by 35 µl of saline was performed, with substances separated by a bubble within one syringe. Behavioral measurement followed 1 h, 2 h, and 4 h after the second i.t. administration. The average values from the hind paws of individual animals were averaged in the experimental groups.

#### Intrathecal catheter implantation

Similar to our previous works^29–31^, intrathecal catheters were implanted between the L3 and L4 vertebrae. Polyethylene tubing PE-5 and PE-10 were connected and glued together to be accessible for the drug administration. The catheter implantation was performed under deep anesthesia of 3% isoflurane. Through a longitudinal incision on top of lumbar L3 and L4 the upper lumbar vertebrae were exposed. The L3 vertebra was gently lifted, and the PE-5 catheter end, filled with saline, was placed into the subarachnoid space and fixed to the spine with super glue. The other catheter end was exposed on head and heat-sealed. The animals were observed for neurological deficits after the catheter implantation. None of the animals used in the experiments showed any neurological deficits. Animals were left to recover in their cages for at least 5 days before testing. The position of the catheters was verified visually by a dye injection at the end of each experiment.

### Statistical analysis

Data are expressed as mean ± SEM, most of which were normalized to the control/baseline value. Statistical analysis was calculated using GraphPad Prism 10 software. For statistical comparisons, the nonparametric tests (Friedman, Kruskal-Wallis) were used for data sets with control without deviation. The t-test, one-way ANOVA, and repeated measures (RM) one-way ANOVA were used for data analysis with normal distribution. P-value < 0.05 was considered statistically significant.

### Materials

All chemicals used for extracellular solutions were of analytical grade and were purchased from Sigma Aldrich (St. Louis, MO, USA) and Tocris Bioscience (Bristol, UK). Anandamide and 20:4-NAPE (Avanti Polar Lipids, Alabaster, AL, USA), LEI-401 (Cayman Chemical, Ann Arbor, MI, USA), PF514273, SB366791 and capsaicin (Tocris Bioscience) were dissolved in DMSO, which always had a concentration of < 0.1% in the final solution.

## Results

### In vivo, intrathecal administration of 20:4-NAPE induced mechanical analgesia and allodynia depending on the concentration

**Fig. 1:**
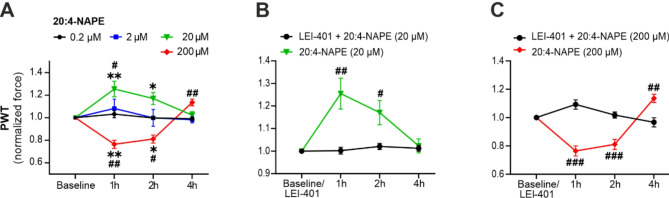
Intrathecal treatment with 20:4-NAPE has antinociceptive and pronociceptive effects on mechanical sensitivity based on concentration and NAPE-PLD activation. (**A**) The PWT to mechanical stimuli measured by the von Frey test after i.t. administration of four concentrations of 20:4-NAPE (0.2 µM, 2 µM, 20 µM, and 200 µM) was normalized to the baseline before i.t. treatment. Administration of 20:4-NAPE at a lower concentration (20 µM) increased the PWT, while a higher concentration (200 µM) decreased the PWT. (**B**) Pretreatment with the NAPE-PLD inhibitor LEI-401 (10 µM, i.t.) prevented the PWT increase induced by 20 µM 20:4-NAPE. (**C**) Pretreatment with LEI-401 (10 µM, i.t.) prevented the PWT decrease induced by 200 µM 20:4-NAPE. The data after 20 and 200 µM 20:4-NAPE application in (**B**,**C**) are the same as in (**A**). The statistical analysis: non-parametric Kruskal-Wallis test vs. baseline, one-way ANOVA with Dunnett’s test vs. 0.2 µM 20:4-NAPE (**A**); t-test vs. LEI-401 (**B**,**C**); criteria for significance ∗*p* < 0.05, ∗∗*p* < 0.01 vs. baseline before i.t. treatment, ^#^*p* < 0.05, ^##^*p* < 0.01, and ^###^*p* < 0.001 vs. 0.2 µM 20:4-NAPE or LEI-401 treatment.

The impact of i.t. administration of 20:4-NAPE on mechanical sensitivity was examined in rats in vivo. Four concentrations, ranging from 0.2 µM to 200 µM, were evaluated in behavioral experiments testing mechanical threshold. The paw withdrawal threshold (PWT) was not altered by the administration of 0.2 µM (*n* = 6) or 2 µM 20:4-NAPE (*n* = 8) between 1 h and 4 h after the i.t. injection (Fig. 1A). Higher concentration of 20:4-NAPE (20 µM) significantly increased the withdrawal threshold 1 h (*n* = 8, *p* < 0.01) and 2 h (*p* < 0.05) after injection, suggesting analgesic effect. The PWT returned to the baseline value 4 h after the treatment. In contrast, further increase in the 20:4-NAPE concentration to 200 µM decreased PWT at 1 h (*n* = 12, *p* < 0.01) and 2 h (*p* < 0.05) after the injection, implying the presence of allodynia. These results indicate that increased levels of 20:4-NAPE may have opposite effects on pain sensitivity, based on the concentration.

To determine whether the production of AEA by NAPE-PLD enzymatic activity is involved in the observed 20:4-NAPE-mediated in vivo effects, the pretreatment with the NAPE-PLD inhibitor, LEI-401 (10 µM, i.t.), was evaluated. When NAPE-PLD inhibitor was applied alone, it did not affect the PWT 1 h after the injection (Baseline: 66.8 ± 1.5 g, LEI-401: 66.5 ± 1.3 g, *n* = 5). Subsequent co-administration of LEI-401 followed by 20:4-NAPE (20 µM, i.t.) did not alter the PWT (Fig. 1B). Thus, the antinociceptive effect of 20 µM 20:4-NAPE was prevented by LEI-401 pretreatment. In the second experiment the LEI-401 pretreatment alone also did not affect the PWT 1 h after the administration (Baseline: 77.8 ± 4.3 g, LEI-401: 76.7 ± 2.4 g, *n* = 4). Subsequent co-administration of LEI-401 followed by 20:4-NAPE (200 µM, i.t.) did not change the PWT (Fig. 1C). The allodynia induced by 200 µM 20:4-NAPE was completely blocked by LEI-401 pretreatment. Thus both antinociceptive and pronociceptive effects elicited by the 20:4-NAPE i.t. administration were dependent on NAPE-PLD activation.

### Concentration-dependent effect of 20:4-NAPE application on DRG neuron excitability

**Fig. 2:**
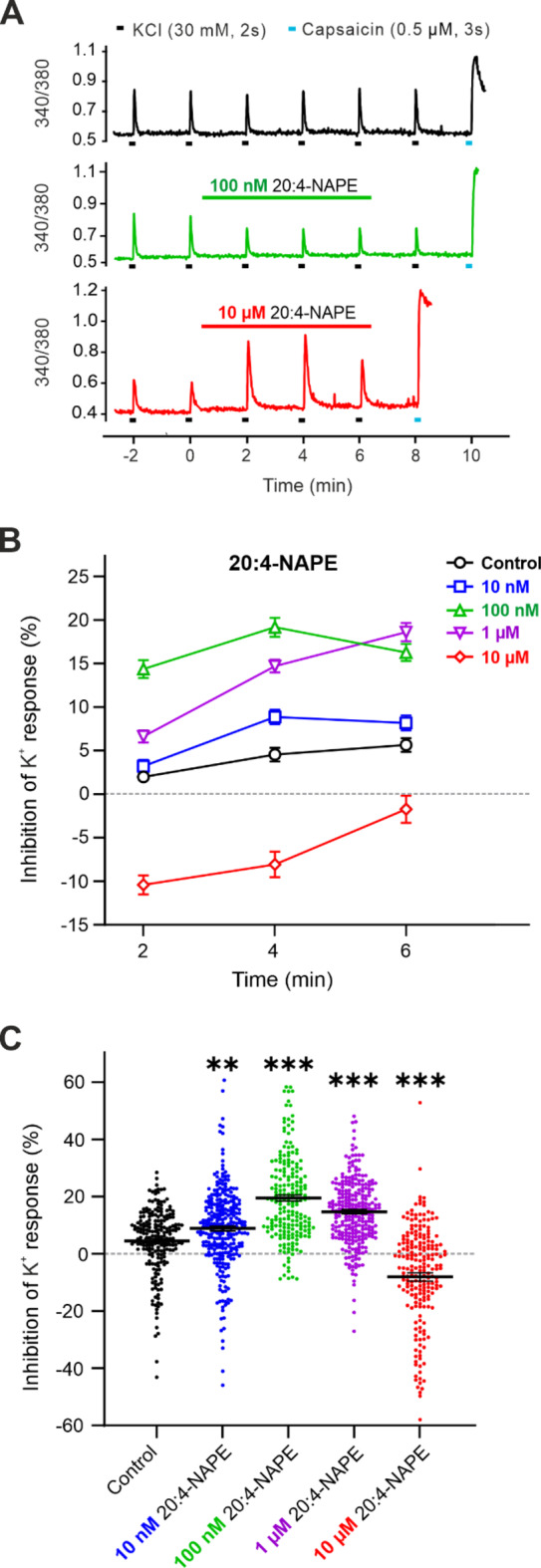
The application of low concentrations of 20:4-NAPE decreased the excitability of DRG neurons, while higher concentration evoked the opposite effect. (**A**) A native measurement of changes in the intracellular Ca^2+^ concentration in single neurons under control conditions was conducted during repeated application of KCl (30 mM, 2 s) at 2 min intervals (black line). The application of 100 nM 20:4-NAPE (6 min, green line) decreased the amplitude of the Ca^2+^ transient (340/380). The application of a high 10 µM concentration of 20:4-NAPE (6 min, red line) increased the amplitude. Capsaicin (0.5 µM, 3 s) applied at the end of the experiment evoked robust response. (**B**) Repeated application of KCl resulted in a slight inhibition of K^+^-induced Ca^2+^ responses in DRG neurons (*n* = 211). The concentration-dependent effect of 20:4-NAPE application (6 min) on the control K^+^-induced Ca^2+^ responses was evaluated in a range of concentrations, comprising 10 nM (*n* = 288, *p* < 0.01 at the 4th min), 100 nM (*n* = 206, *p* < 0.001 at the 2nd, 4th and 6th min), 1 µM (*n* = 269, *p* < 0.001 at the 2nd, 4th and 6th min), and 10 µM (*n* = 211, *p* < 0.001 at 2nd, 4th and 6th min). Statistical significance is shown relative to the Control (black line). (**C**) A representation of the dual effect induced by 20:4-NAPE at the 4th min. A statistically significant difference was detected in the inhibitory and excitatory effects induced by various concentrations of 20:4-NAPE compared to the Control (black). All cells analysed are depicted. One-way ANOVA (Dunnett’s test) was used for statistical analysis, with criteria for significance ∗∗*p* < 0.01, ∗∗∗*p* < 0.001.

The impact of 20:4-NAPE on the excitability of primary sensory neurons was investigated in rat DRG cultures. Nociceptive DRG neurons were repeatedly stimulated with a high concentration of KCl (30 mM) every 2 min, and changes in intracellular Ca^2+^ concentration were measured according to the experimental protocol (see Methods). The control Ca^2+^ transient amplitude was averaged from the 1st and 2nd K^+^-induced responses just before the drug application and was set to 0%. The inhibition of the K^+^-induced Ca^2+^ transients amplitude was then calculated as a percentage of the control stimulations and presented as positive values in individual graphs. During the repeated excitation of the DRG neurons under the control conditions, the amplitude of the Ca^2+^ transients showed a small but significant reduction. The inhibition of the K^+^-evoked response at the 2nd min was 2.0 ± 0.53% (*p* < 0.001), at the 4th min 4.5 ± 0.79% (*p* < 0.001) and at the 6th min of the experiment 5.6 ± 0.79% (*p* < 0.001, Fig. 2A,B). A potent TRPV1 agonist capsaicin was applied at the end of this control experiment. All 211 neurons were tested with capsaicin (0.5 µM, 3 s), and the Ca²⁺ response was elicited in 181 of these neurons, representing 86% of the total. This confirmed that our study was conducted preferentially on the population of nociceptive DRG neurons due to the 40 μm strainer application during the culture preparation, and the vast majority of them expressed functional TRPV1 and responded to the capsaicin application with Ca^2+^ transient.

Based on this experimental protocol, the effect of four different concentrations of 20:4-NAPE (10 nM, 100 nM, 1 µM, and 10 µM) on the excitability of DRG neurons was tested in separate experiments. Application of 20:4-NAPE (6 min) at lower concentrations significantly inhibited the K^+^ response at the 2nd min of the application (10 nM, *p* < 0.001; 100 nM, *p* < 0.001; and 1 µM, *p* < 0.001), at the 4th min (10 nM, *p* < 0.001; 100 nM, *p* < 0.001; 1 µM, *p* < 0.001), and at the 6th min (10 nM, *p* < 0.001; 100 nM, *p* < 0.001; 1 µM, *p* < 0.001). In contrast, the application of the highest 10 µM concentration of 20:4-NAPE significantly enhanced K^+^ response in the 2nd min (*p* < 0.001) and the 4th min (*p* < 0.001). At the end (6th min) of the 20:4-NAPE application, the response returned close to the control level present before the 20:4-NAPE treatment (Fig. 2A, B).

Compared to the control situation at the respective times of the application, lower concentrations of 20:4-NAPE significantly inhibited the K^+^ response. This effect was observed at 10 nM, 100 nM, and 1 µM concentrations, with a stronger inhibition observed during 100 nM 20:4-NAPE application. In comparison, the higher 10 µM concentration of 20:4-NAPE significantly potentiated the K^+^-evoked Ca^2+^ transients (Fig. 2B, C). Application of 20:4-NAPE produced concentration-dependent effects on nociceptive DRG neurons. At lower concentrations, 20:4-NAPE decreased the excitability of DRG neurons. Conversely, the high concentration of 20:4-NAPE (10 µM) had the opposite effect.

### Concentration-dependent effect of Anandamide application on DRG neuron excitability

**Fig. 3:**
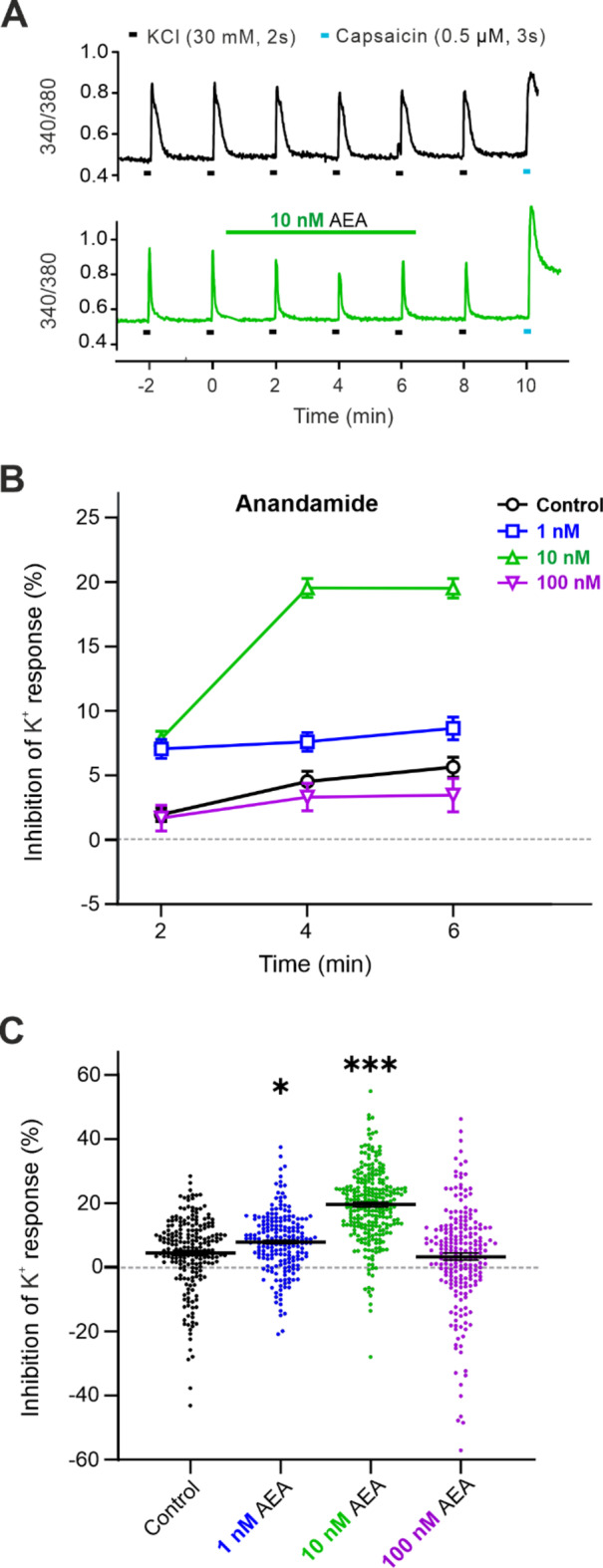
The concentration-dependent inhibition of the DRG neurons excitability by exogenous AEA application. (**A**) Repeated application of KCl (30 mM, 2 s) at 2 min intervals induced the increase of the intracellular level of Ca^2+^ in a single neuron under control conditions (black line). The application of 10 nM AEA (6 min, green line) decreased the amplitude of the 340/380. Capsaicin (0.5 µM, 3 s) was applied at the end of the experiment. (**B**) Repeated application of KCl in control conditions resulted in slight inhibition of K^+^-induced Ca^2+^ responses in DRG neurons (*n* = 211). AEA at a concentration of 1 nM further inhibited the control K^+^-induced response at the 2nd min (*n* = 202, *p* < 0.001) and 4th min (*p* < 0.05) of the application. The inhibitory effect of 10 nM AEA increased continuously until the 4th min and persisted until the 6th min (*n* = 263, *p* < 0.001 at 2nd, 4th, and 6th min). Application of 100 nM AEA (*n* = 218) did not alter the control K^+^-induced responses. Statistical significance was calculated relative to the Control (black line). (**C**) Depiction of a representative inhibitory effect induced by AEA at the 4th min. The application of 1 nM and 10 nM AEA significantly increased the inhibition compared to the control conditions (black). One-way ANOVA (Dunnett’s test) was used for statistical analysis, with criteria for significance ∗*p* < 0.05, ∗∗∗*p* < 0.001.

Analogous to the experiments with 20:4-NAPE, we examined the impact of direct application of AEA on the excitability of DRG neurons. In order to ascertain the effects of different AEA concentrations, separate experiments were conducted at three lower concentrations within the nM range (1 nM, 10 nM, and 100 nM; 6 min). AEA in higher concentrations has been demonstrated to excite DRG neurons directly^32,33^, and therefore, the effects of 10 µM AEA were tested separately (see Fig. 6). AEA at a concentration of 1 nM significantly increased the inhibition of the K^+^-induced response at the 2nd min of the application (*p* < 0.001), with the level of this inhibition remaining unchanged until the end of the AEA application (*p* < 0.001 at the 4th and 6th min; Fig. 3A,B). The inhibitory effect of 10 nM AEA (*p* < 0.001) was comparable to that of 1 nM AEA at the 2nd min of the application. However, this effect was further significantly enhanced at the 4th min of the application (*p* < 0.001) and persisted until the end of the application at the 6th min (*p* < 0.001). AEA at the concentration of 100 nM did not elicit the inhibition of the K^+^-induced Ca^2+^ response at the 2nd min of the application. Nevertheless, a slight inhibition was observed at the 4th min (*p* < 0.001), and the level remained similar until the end of the application (*p* < 0.001 at the 6th min).

The lowest 1 nM AEA concentration significantly inhibited the K^+^-evoked response compared to the control inhibition of K^+^ responses at the 2nd and 4th min of the application. The tenfold higher 10 nM AEA concentration elicited the most pronounced inhibition during the whole AEA application. The response of a hundredfold higher 100 nM AEA concentration did not differ from the control K^+^ response during the whole AEA application (Fig. 3B,C).

The application of the exogenous AEA resulted in a concentration-dependent inhibition (1 nM and 10 nM) of the excitability of nociceptive DRG neurons, with the greatest effect observed at 10 nM. However, the further elevation of the AEA concentration to 100 nM did not affect the excitability of DRG neurons induced by potassium ions.

### Blocking NAPE-PLD prevents the 20:4-NAPE-induced inhibition/excitation but does not affect AEA effects

**Fig. 4:**
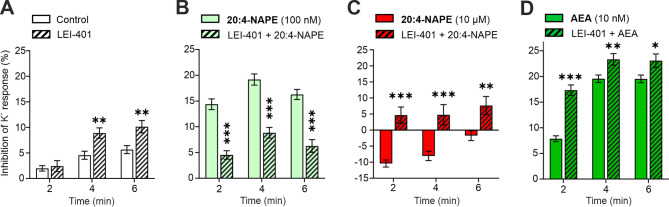
NAPE-PLD inhibitor, LEI-401, prevents the 20:4-NAPE-induced inhibition and excitation of DRG neuron excitability. (**A**) Incubation (1 µM, 2 h) and subsequent acute application of LEI-401 (1 µM) increased the inhibition of the K^+^ response in DRG neurons (*n* = 110) versus Control (*n* = 211). (**B**) Co-application of LEI-401 (1 µM) and 20:4-NAPE (100 nM, *n* = 267), which followed the incubation of cells with LEI-401 (1 µM, 2 h), prevented the inhibitory effect of 100 nM 20:4-NAPE application (*n* = 206). (**C**) Excitatory effect induced by 10 µM 20:4-NAPE application (*n* = 211) was prevented by pre-incubation with LEI-401 (1 µM, 2 h) and subsequent co-application of LEI-401 (1 µM) and 20:4-NAPE (10 µM, *n* = 62). (**D**) Co-application of LEI-401 (1 µM) and AEA (10 nM, *n* = 275) after cells incubation with LEI-401 (1 µM, 2 h) increased the inhibitory effect of AEA (10 nM) application alone (*n* = 263). Statistical analysis: t-test, Control vs. LEI-401, NAPE vs. NAPE + LEI-401, AEA vs. AEA + LEI-401, ∗*p* < 0.05, ∗∗*p* < 0.01, ∗∗∗*p* < 0.001.

A key enzyme involved in AEA synthesis, NAPE-PLD, is expressed in DRG neurons^34^. In our experiments, we tested whether NAPE-PLD mediates AEA production from 20:4-NAPE to subsequently induce the inhibitory and excitatory effects on DRG neuron excitability. Incubation of cells (2 h) with subsequent acute application of LEI-401 (1 µM), a NAPE-PLD inhibitor, slightly increased the inhibition of the K^+^ response in DRG neurons (Fig. 4A) compared to the control experiment without NAPE-PLD inhibition. Although LEI-401 treatment itself had an effect, it also prevented the inhibitory effect of 100 nM 20:4-NAPE application (Fig. 4B) and the excitatory effect of 10 µM 20:4-NAPE application (Fig. 4C). In contrast to 20:4-NAPE, the inhibitory effect of 10 nM AEA application was not prevented by LEI-401 treatment. However, co-application of LEI-401 with 10 nM AEA did not prevent but increased the inhibitory effect compared to AEA applied alone (Fig. 4D). This enhancement of the excitability inhibition may reflect the sub-additive effect of LEI-401 and 10 nM AEA. These results suggest that the production of AEA from 20:4-NAPE via NAPE-PLD likely mediated both the inhibitory and excitatory effects on the excitability of DRG neurons.

### CB_1_ and TRPV1 receptor-mediated mechanisms of action of 20:4-NAPE and AEA

In order to investigate the mechanism underlying the effects mediated by the application of 20:4-NAPE and exogenous AEA, the main AEA target receptors CB_1_ and TRPV1 were blocked by the antagonists PF514273 or SB366791, respectively.

#### Activation of the CB_1_ receptor mediated the inhibitory effects of 20:4-NAPE and AEA application

**Fig. 5:**
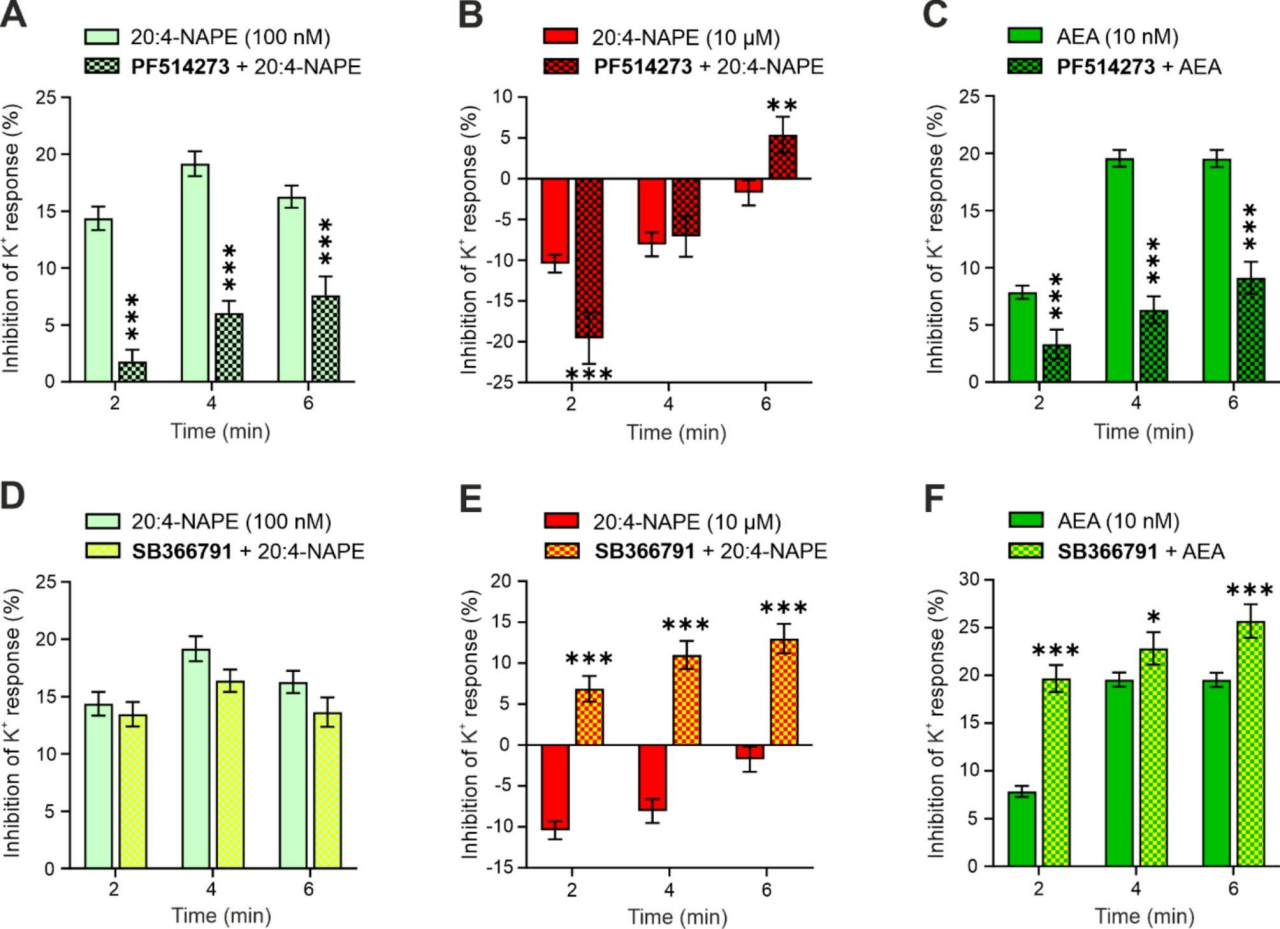
The inhibitory effect of 20:4-NAPE and AEA was mediated by CB_1_ receptor activation, whereas TRPV1 activation mediated the 20:4-NAPE-induced excitatory effect. (**A**) The inhibition of K^+^ responses evoked by 100 nM 20:4-NAPE (*n* = 206) was prevented by the co-application of the CB_1_ antagonist PF514273 (*n* = 121). (**B**) The excitatory effect of 10 µM 20:4-NAPE (*n* = 211) was not blocked by PF514273 treatment (*n* = 96). (**C**) The inhibitory effect of low 10 nM AEA (*n* = 263) concentration was prevented by the PF514273 treatment (*n* = 115). (**D**) The inhibitory effect induced by the 100 nM 20:4-NAPE (*n* = 206) application was not altered by the TRPV1 antagonist SB366791 co-application (*n* = 189). (**E**) The excitatory effect of 10 µM 20:4-NAPE (*n* = 211) was prevented by the SB366791 treatment (*n* = 113). (**F**) The inhibitory effect of the low 10 nM AEA (*n* = 263) concentration was enhanced by the SB366791 treatment (*n* = 120). The statistical analysis was conducted using a t-test, with the following significance levels: ∗ *p* < 0.05, ∗∗*p* < 0.01, and ∗∗∗*p* < 0.001.

The following three experiments investigated the role of the CB_1_ receptor activation in the effects evoked by the application of 20:4-NAPE and AEA. First, the impact of the CB_1_ receptor inhibition on the inhibitory effect of 100 nM 20:4-NAPE was tested. Before the experiment, the CB_1_ receptor antagonist PF514273 was applied alone. The inhibition of the K^+^ response elicited by PF514273 (400 nM, *n* = 174, 2nd min: 3.3 ± 1.1, 4th min: 5.2 ± 1.1, 6th min: 7.0 ± 1.2) was not significantly different from the control (Fig. 2B). This indicates that CB_1_ receptor inhibition did not alter the K^+^ induced excitability of DRG neurons. Pretreatment with CB_1_ receptor antagonist PF514273 (400 nM, 4 min) and subsequent co-application of PF514273 with 20:4-NAPE (100 nM, 6 min) prevented the inhibitory effect of 100 nM 20:4-NAPE (Fig. 5A). This suggests that the inhibitory effect of 20:4-NAPE on DRG neuron excitability was mediated by CB_1_ receptor activation.

Second, the effect of the CB_1_ receptor inhibition on the excitatory effect induced by 10 µM 20:4-NAPE was assessed. Pretreatment with PF514273 (400 nM, 4 min) followed by co-application of PF514273 with 20:4-NAPE (10 µM, 6 min) had a time-dependent effect on DRG excitability. At the 2nd min of co-application, the excitatory effect of 20:4-NAPE was significantly higher compared to the situation without the CB_1_ antagonist. At the 4th min, the excitatory effect of 20:4-NAPE was not affected, and at the 6th min of co-application, the effect of 20:4-NAPE switched to inhibitory (Fig. 5B). When the CB_1_ receptors were blocked, the excitatory effect of 20:4-NAPE was increased, probably due to reduced inhibition mediated by CB_1_ receptors activated by the endogenously produced AEA from 20:4-NAPE. However, this effect was time-dependent, which may reflect TRPV1 receptors desensitization or the decrease in AEA concentration needed to activate TRPV1 receptors.

Third, the role of CB_1_ antagonist was tested on the inhibitory effect of low concentration of exogenous AEA (10 nM). Pretreatment with PF514273 (400 nM, 4 min) followed by co-application of PF514273 with AEA (10 nM, 6 min) prevented the inhibitory effect of AEA on the DRG excitability (Fig. 5C). Consequently, the inhibitory effect of AEA was mediated by CB_1_ receptor activation.

#### Activation of the TRPV1 receptors mediated the excitatory effect of 20:4-NAPE

First, the effect of the TRPV1 antagonist SB366791 was tested alone. The Ca^2+^ transients induced by the K^+^ with the SB366791 present (1 µM, *n* = 103) were different from the control (Fig. 2B) only at the 2nd min (6.0% ± 1.4, *p* < 0.01, 4th min: 4.2% ± 1.6, 6th min: 7.0% ± 1.4). This indicates that TRPV1 receptors do not play an important role in this type of DRG neurons activation under the control conditions.

The importance of the TRPV1 activation in the effects evoked by the application of 20:4-NAPE and AEA was evaluated in three experiments. Pretreatment with the TRPV1 antagonist SB366791 (1 µM, 4 min) and subsequent co-application of SB366791 with 20:4-NAPE (100 nM, 6 min) did not alter the inhibitory effect of 100 nM 20:4-NAPE when applied alone (Fig. 5D). The mechanism of 20:4-NAPE-induced inhibition did not involve the activation of TRPV1.

Second, the impact of TRPV1 antagonist on the excitatory effect induced by 10 µM 20:4-NAPE was evaluated. Pretreatment with SB366791 (1 µM, 4 min) followed by co-application of SB366791 with 20:4-NAPE (10 µM, 6 min) prevented the excitatory effect of 10 µM 20:4-NAPE on DRG neurons throughout the entire period of 20:4-NAPE application (Fig. 5E) and induced robust inhibition.

Third, the role of TRPV1 activation on the inhibitory effect of exogenously added AEA (10 nM) was investigated. Pretreatment with SB366791 (1 µM, 4 min) followed by co-application of SB366791 with AEA (10 nM, 6 min) did not prevent the inhibitory effect of 10 nM AEA application. Furthermore, the AEA-induced inhibition of K^+^ responses in DRG neurons increased significantly (Fig. 5F). This indicates that the inhibition of TRPV1 enhanced the CB_1_ receptor activation-mediated inhibitory effect of AEA.

### Application of high concentration of AEA induced Ca^2+^ transients via TRPV1 activation in DRG neurons

Higher concentrations of AEA are known to induce Ca^2+^ transients in DRG neurons^32,33^. Therefore, we investigated the underlying mechanism of AEA-induced excitation of DRG neurons by repeated application of high AEA concentration, on which antagonists of CB_1_ and TRPV1 receptors were tested.

**Fig. 6:**
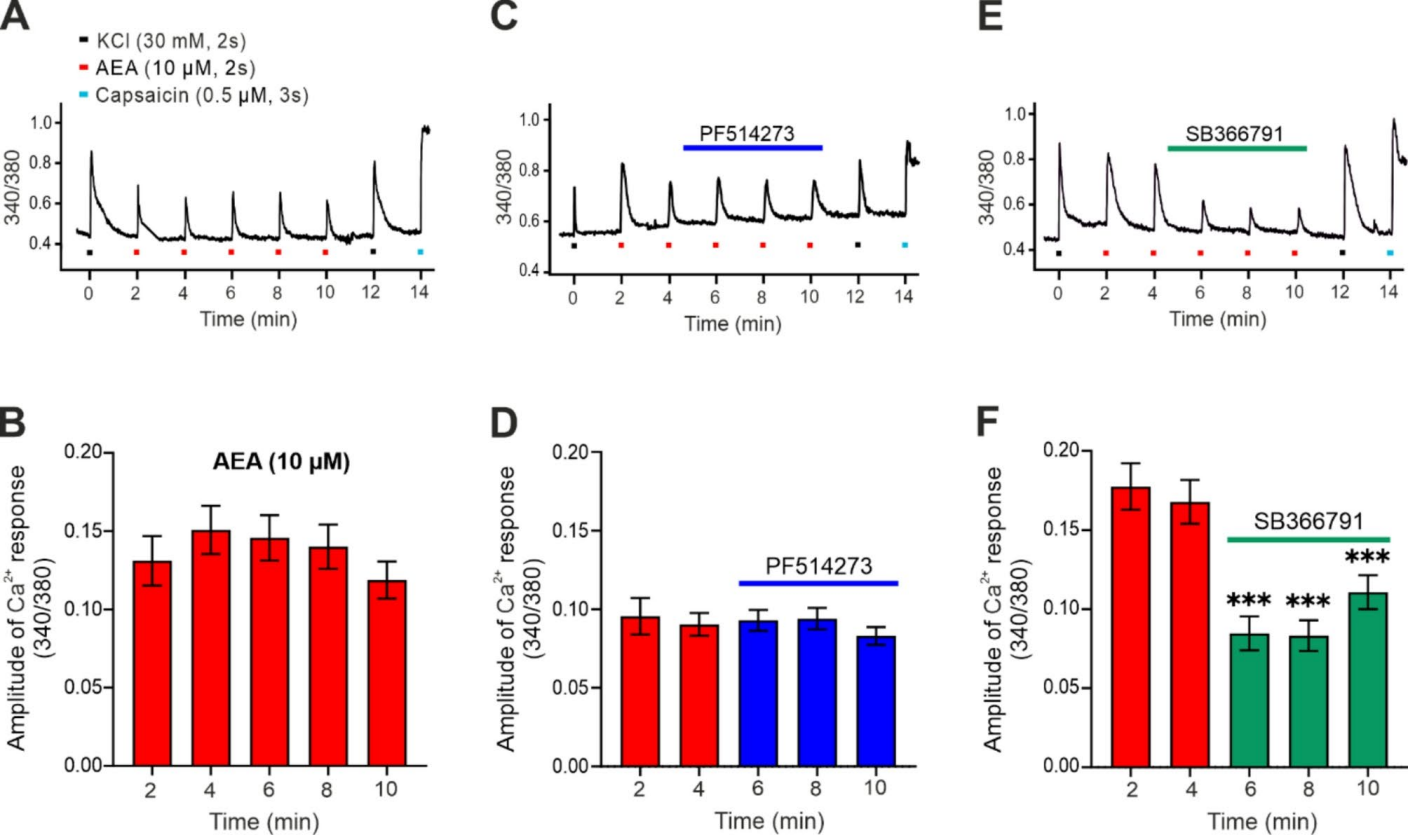
AEA-induced Ca^2+^ transients in DRG neurons were diminished by TRPV1 inhibition. (**A**) A series of five consecutive applications of a high concentration of AEA (10 µM, 2 s) was employed to induce Ca^2+^ transients in nociceptive DRG neurons. KCl (30 mM, 2 s) was applied before and after the AEA applications. At the end of the experiment, capsaicin (0.5 µM, 3 s) was used. All applications were conducted at 2-min intervals, as demonstrated on a single neuron recordings. (**B**) Five consecutive applications of AEA (10 µM, 2 s, *n* = 59) evoked Ca^2+^ transients of similar amplitude. (**C**) The recording from a single DRG neuron shows a prolonged application of the CB_1_ receptor antagonist PF514273 performed after two brief AEA applications. (**D**) This application of PF514273 did not change the AEA-induced amplitude of Ca^2+^ transients (*n* = 94). (**E**) The recording from a single DRG neuron shows a prolonged application of the TRPV1 antagonist SB366791 performed after two brief applications of AEA. (**F**) This application of SB366791 significantly decreased the AEA-induced amplitude of Ca^2+^ transients in the whole period of antagonist application (*n* = 101). The statistical analysis was conducted using a RM one-way ANOVA (Dunnett’s test), with the following significance levels: ∗∗∗*p* < 0.001.

Anandamide (10 µM, 2 s, 2-min interval) was applied five times, while KCl (30 mM, 2 s) was used to test the excitability of neurons before and after the AEA application. At the end of the protocol, capsaicin (0.5 µM, 3 s) was also applied to identify cells expressing TRPV1 (Fig. 6A). Five consecutive applications of AEA evoked Ca^2+^ transients of similar amplitude (Fig. 6B). The CB_1_ antagonist was tested in different experiments, where the acute application of PF514273 (400 nM, 6 min) did not alter the amplitude of Ca^2+^ responses elicited by brief AEA applications (Fig. 6C,D). In another set of experiments, the TRPV1 antagonist SB36791 was tested in the same fashion. Application of SB36791 (1 µM, 6 min) significantly decreased the Ca^2+^ response amplitude elicited by AEA application (Fig. 6E,F). These results demonstrate that the increase in intracellular Ca^2+^ induced by 10 µM AEA application in nociceptive DRG neurons involved activation of TRPV1 but not CB_1_ receptors.

## Discussion

Our study is the first in vivo evaluation of the analgesic effect of intrathecal administration of 20:4-NAPE. The lower concentration of 20:4-NAPE (20 µM) elicited hyposensitivity to mechanical stimuli, whereas the higher concentration (200 µM) caused mechanical allodynia. Both in vivo effects were dependent on NAPE-PLD activity, suggesting that local AEA production from 20:4-NAPE is involved in the modulation of tactile sensitivity.

Alterations in the excitability of DRG neurons contribute to hypersensitive states such as allodynia and hyperalgesia and to spontaneous pain that may occur in chronic pain conditions. In our work, analogous to the impact on tactile sensitivity in vivo, 20:4-NAPE modulated the excitability of DRG neurons in vitro. Application of 20:4-NAPE elicited the concentration-dependent effect on the excitability of nociceptive DRG neurons. The excitability was decreased by the lower concentrations of 20:4-NAPE (from 10 nM to 1 µM) and increased by the application of the high concentration (10 µM). Both excitatory and inhibitory modulatory effects of 20:4-NAPE were prevented by NAPE-PLD antagonist (LEI-401), suggesting that enzymatic activity of NAPE-PLD present in DRG culture was needed. These results also imply that CB_1_ and TRPV1 receptors are activated after 20:4-NAPE application due to endogenous AEA production and their direct activation by 20:4-NAPE is unlikely. Externally added AEA had a similar impact on DRG neuron excitability. Lower concentrations of AEA (from 1 nM to 10 nM) decreased the K^+^-induced neuronal excitation, whereas high concentration of AEA (10 µM) produced excitation similar to that evoked by the K^+^ solution. Inhibition of the CB_1_ receptor by PF514273 prevented the 20:4-NAPE- or AEA-induced decrease in excitability, whereas TRPV1 inhibition by SB366791 prevented the increase in DRG neuron excitability.

### Antinociceptive effect of spinal 20:4-NAPE administration converted to allodynia at high concentration

The AEA-induced analgesia was recognized to be underlined by a peripheral mechanism via CB_1_ receptor activation in the animal model of neuropathic pain, while increased AEA levels were achieved with a peripherally restricted FAAH inhibitor (URB937)^2^. In comparison, AEA-induced analgesia mediated by spinal mechanisms has been shown to alleviate neuropathic or inflammatory pain when administered intrathecally. Both CB_1_ and TRPV1 receptors have been implicated in the underlying mechanisms^35–38^. In our experiments, we demonstrated in naïve animals that the intrathecal treatment with 20:4-NAPE elicited a dose-dependent effect via NAPE-PLD activation, as lower concentrations induced antinociceptive and high exerted the pro-nociceptive effects. These in vivo effects are consistent with those observed in nociceptive DRG neurons, while we did not report increased activation after 20:4-NAPE application in spinal cord slices^18^. Presynaptic CB_1_ and TRPV1 receptor activation is likely to be involved in the underlying mechanism. Compared to the situation in DRG neuronal cultures, the production of AEA from 20:4-NAPE could also be mediated in other cell types, such as glial cells, due to the presence of NAPE-PLD^34,39^. Our experiments strongly suggest that the dosage of 20:4-NAPE should be carefully determined to achieve analgesia. Excessive production of AEA by NAPE-PLD in the spinal cord may lead to unfavorable pro-nociceptive effects. Altered expression of endocannabinoidome components, including AEA synthesizing and degrading enzymes and target receptors induced by pathological pain conditions, should also be considered in any possible pain treatment application^9,40^. Previous evidence indicates that peripheral inflammation may induce a switch of the mechanism underlying the inhibition of spinal excitatory synaptic transmission at first nociceptive synapses evoked by 20:4-NAPE application from the CB_1_ dependent one to both CB_1_/TRPV1 mediated^18,19^. The potential use of AEA-modulating drugs for pain treatment may be quite complex. Specific changes in the endocannabinoidome homeostasis in various pathological pain states may alter the mechanisms of endocannabinoid-mediated analgesia, which remains incompletely understood.

### 20:4-NAPE induced modulation of nociceptive signaling in the spinal cord

In our previous experiments using spinal cord slices, we have suggested that 20:4-NAPE may be better suited for analgesic purposes, compared to AEA application, due to its consistent inhibitory effects on nociceptive synaptic transmission at the first nociceptive synapses in the spinal cord dorsal horn^17–19^. The hypothesis is that local production of AEA from NAPE, leading to microdomain concentration gradients and activation of surrounding receptors, would be more efficient and have different effects compared to flooding the preparation with exogenous AEA. The presynaptic side of these dorsal horn synapses is formed by central endings of DRG neurons, and neurotransmitter release may be regulated by CB_1_ and TRPV1 receptors co-expressed on these DRG neurons. However, our in vivo experiments in this work demonstrated that while intrathecal application of low concentration of 20:4-NAPE had strong analgesic effect, the high concentration was pro-nociceptive. This corresponds to previous findings with direct AEA application. AEA at low concentrations inhibited potassium- and capsaicin-evoked release of pronociceptive calcitonin gene-related peptide (CGRP) in the spinal cord dorsal horn^35^, but at high concentrations, AEA directly evoked release of CGRP and substance P^33^. These actions parallel activation of CB_1_ receptors leading to decreased transmitter release and activation of TRPV1 channels, increased Ca^2+^ influx and enhanced glutamate release^13–15^. Activation of TRPV1 receptors with high capsaicin concentration, elicited action potentials (APs) in superficial dorsal horn neurons, even while preventing evoked glutamate release by the dorsal root stimulation^13,41^. In this process, a down-regulation of voltage-activated Ca^2+^ channels (VACCs) by dephosphorylation via calcineurin following TRPV1 activation may play a role^42^. In addition, using a voltage-sensitive dye, it has been shown that TRPV1 activation also reduced the presynaptic excitation evoked by dorsal root stimulation of nociceptive fibers^43^. Notably, electrical stimulation of solitary tract afferents revealed that asynchronous glutamate release triggered long-lasting AP firing on the postsynaptic neuron, and this ability was mediated by TRPV1^44^. Taken together, TRPV1-mediated facilitation of nociceptive transmission in the spinal cord dorsal horn likely contributes to enhanced pain signaling, while robust TRPV1 activation prevent excessive nociceptive transmission.

### Impact of 20:4-NAPE on the excitability of DRG neurons

The pseudo-unipolar DRG neurons express CB_1_ and TRPV1 receptors on both the proximal and distal axonal process, that form the presynaptic ending in the spinal cord and the peripheral nerve ending in the periphery^45,46^. While the DRG neurons in acute cell culture usually lack these processes, they are used to study the function of receptors expressed in these endings in vivo. In the present study, we have used cultured DRG neurons to further investigate the 20:4-NAPE-mediated modulation of DRG neurons excitation. Both 20:4-NAPE- and externally added AEA at lower concentrations inhibited the excitability of DRG neurons via CB_1_ receptor activation, whereas higher concentrations increased it via TRPV1 activation. Our findings are consistent with previous reports of AEA-induced effects in DRG neurons. AEA at low concentrations activated the CB_1_ receptors in DRG neurons to inhibit Ca^2+^ transients induced by electrical field stimulation^33^, and to decrease the calcitonin gene-related peptide (CGRP) release in DRG culture^47^. On the other hand, the high concentrations of AEA induced Ca^2+^ transients, potentiated CGRP release and increased spontaneous action potentials generation via the TRPV1 activation^33,47–49^. In comparison, Evans et al. (2004) showed that a single concentration of AEA (1 µM) induced a dual effect on the potassium-induced Ca^2+^-mediated response, with the observed decrease or increase in excitability being segregated by DRG neuron body size. In these experiments, the inhibitory effect depended on the activation of the G_i/o_ protein pathway, which is also involved in the signaling downstream of CB_1_ receptor activation^50^. Taken together, previous studies and our current results thus strongly support the evidence for a concentration-dependent and CB_1_- or TRPV1-mediated effect of AEA on nociceptive DRG neurons. However, the AEA-induced effect may also be body size dependent, pointing to the heterogeneity of DRG neurons with differential expression of CB_1_ and TRPV1 receptors that adds complexity to the effect induced by AEA^10,51^. In our experiments, we studied a population of small diameter neurons (< 40 μm), majority of which showed responses to TRPV1 receptors activation by capsaicin. Thus, the local concentration of AEA produced from 20:4-NAPE and the exclusive expression or spatial distribution of CB_1_/TRPV1 receptors^52^ in the subpopulations of DRG neurons are the essential factors that delineate the effect of endocannabinoids on the excitability of DRG neurons.

While the application of high concentrations of both 20:4-NAPE and AEA induced excitation, there were some differences. Application of 20:4-NAPE did not change the baseline Ca^2+^ level and potentiated the K^+^-induced Ca^2+^ transients. However, this potentiation was clearly reduced at the end of the 6 min application time, probably due to desensitization of TRPV1 receptors. Although we cannot also exclude the possibility that the AEA production/concentration was declining with time, it is unlikely as no change at this time period was observed when lower concentrations of 20:4-NAPE were applied. In addition, the excitatory effect of the high concentration of 20:4-NAPE was enhanced during the first K^+^-induced depolarization when CB_1_ receptors were blocked. This suggests that endogenously produced AEA concomitantly activates both CB_1_ and TRPV1 receptors. Application of high concentration of AEA evoked robust Ca^2+^ transients and therefore could not be tested with the K^+^-induced depolarization protocol. These AEA-induced transients did not change their amplitude with repeated application, were not modulated by CB_1_ antagonist application, but were significantly reduced when TRPV1 receptors activation was blocked. These differences suggest that activation of CB_1_ and TRPV1 receptors by 20:4-NAPE and AEA may not be equal and may have different consequences on DRG neuron excitability.

When the NAPE-PLD inhibitor LEI-401^53^ was added alone, in our experiments, the K⁺-induced calcium transients in DRG neurons were slightly suppressed compared to the control conditions. NAPE-PLD is a Ca²⁺-dependent enzyme, so its basal activity under control conditions is likely low. However, the influence of different factors present in the culture media on its activity or some unspecific effects of LEI-401 cannot be excluded. Inhibition of NAPE-PLD may alter the composition of NAPEs in membranes as well as endogenous lipid signaling, including activation of alternative enzymatic pathways. This metabolic imbalance may become more pronounced when DRG neurons are stimulated and accumulate Ca²⁺ ions. The alteration in membrane phospholipids and bioactive lipids composition may potentially affect neuronal excitability also indirectly by modulation of membrane properties, such as membrane fluidity, curvature, and lipid raft organization, and modulate function of ion channels critical for DRG neuron excitability.

Previous studies have demonstrated that a number of endogenous lipids may activate TRPV1 and CB_1_ receptors^9,54^. In our earlier experiments, we found that increased concentrations of 20:4-NAPE lead to increased levels of AEA in spinal cord slices^18^. In the recent study, we have demonstrated that the effects of 20:4-NAPE were dependent on NAPE-PLD activation, further supporting the role of endogenous AEA production via this calcium-sensitive enzyme. Increased activity in DRG neurons leads to increased AEA production, driven by elevated intracellular Ca^2+^ levels^48^. Our results show that the concentration of produced AEA can subsequently have complex effects based on the activation of both CB_1_ and TRPV1 receptors. DRG neurons also express AEA-synthesizing enzymes that are calcium-independent but can be activated by protein kinases^23,24^. At the same time, activation of protein kinases is critical for TRPV1 sensitization and has been implicated in different pathological pain conditions^15,31,55,56^. The role of individual AEA synthesizing enzymes in DRG neurons and their importance for pain modulation in normal and pathological conditions needs further investigation.

## Data Availability

The experimental data are available from the corresponding author on reasonable request.
